# From the Editor’s Desk

**Published:** 2011-10

**Authors:** AJ Brink

**Affiliations:** Cardiovascular Journal of Africa

For the first time, the *Cardiovascular Journal of Africa* has been evaluated for impact factor in the new Journal Citation Report of the ISI.

What is the impact factor and what are its implications? For any journal to have an impact factor, it must be tracked by Thompson Reuters (ISI) for three years. The Journal Citation Index provides a measure of the frequency of which an average article is cited each year in the two years after publication.

The journal’s impact factor does not tell us anything about how widely the journal is read or anything about the quality of any specific research article in the published journal. In fact, the impact factor was intended for librarians as a means of evaluating whether to purchase the title. The impact factor is usually placed prominently on the journal’s website or in the printed journal.

The impact factor for the *Cardiovascular Journal of Africa* for 2010 is 0.708 and the total number of citations was 105. The number of articles in 2008 was 47. The impact factors of other South African and African medical journals are:

*African Journal of AIDS Research* 0.425
*South African Medical Journal* 1.676
*South African Journal of Surgery* 0.233
*South African Journal of Science* 0.596

To increase the value of the impact factor, it is important that every effort be made to cite your articles as widely as possible.

